# Prostate cancer screening with Prostate-Specific Antigen (PSA) testing: A retrospective study

**DOI:** 10.12669/pjms.40.10.8558

**Published:** 2024-11

**Authors:** Olgun Goktas

**Affiliations:** Dr. Olgun Goktas Associate Professor, Uludag University Family Health Center, Nilufer, Bursa, Turkey

**Keywords:** Prostate cancer, Prostate-specific antigen, PSA testing, Screening, Detection

## Abstract

**Objective:**

To evaluate the results of the prostate-specific antigen (PSA) test used in prostate cancer screening.

**Methods:**

This retrospective study was conducted on a total of 1106 male individuals, including 615 people (55.6%) aged 40-54, 379 people (34.3%) aged 55-69, and 112 individuals (10.1%) aged 70 and over. In the study, prostate-specific antigen (PSA) blood test results were performed on a total of 1106 male individuals aged 40 and over registered in the Uludag University Family Health Center, Bursa, Turkey during the 5-years between June 1, 2018, and May 31, 2023, were taken from the database and analyzed. Relationships with sociodemographic characteristics, comorbidities, and risk factors were examined. Data was analyzed using SPSS 25.

**Results:**

About 15.5% of the participants were married, 78.4% were single, and 6% were separated from their spouses or widows. The PSA values of the patients referred to the urologist were found to be significantly different between the ages of 55 and 69. It was determined that the PSA levels of the patients with benign prostatic hypertrophy and prostate cancer were higher than the patients without any finding, and the mean was 6.81±4.39 (p=0.01).

**Conclusion:**

In our study, it is important to diagnose benign prostatic hypertrophy or prostate cancer in patients aged 55-69 years, whose mean PSA levels were 6.81±4.39, and referred to a urologist. As a contribution to the discussions in the literature, we recommend that the patient with symptoms be referred to the urologist after the test request and the joint decision with the family physician.

## INTRODUCTION

Today, it is important that cancer and cancer-related death rates increase in parallel with the risk factors related to globalization and economic growth. According to the GLOBOCAN 2020 cancer incidence and mortality estimates produced by the International Agency for Research on Cancer, the most diagnosed cancers have been determined. In this ranking, breast cancer (11.7%) is the most diagnosed cancer worldwide, followed by lung (11.4%), colorectal (10.0%), prostate (7.3%), and stomach (5.6%) cancers. The fact that prostate cancer ranks fourth is important for the male population.^1^

The rate of cancer is accelerating and because of its diversity, global efforts are needed to contain it. For this purpose, the World Health Organization (WHO) recommends that population screening be carried out within an organized screening program that includes certain basic elements from identification of target populations to treatment, monitoring, and evaluation.^2^

Prostate cancer, which is among the most common cancers in men, is among the most researched cancers. Today, it is seen that only one in every four men diagnosed with cancer in the world is diagnosed with prostate cancer. Despite the increase in the number of patients, prostate cancer-related death rates have decreased by approximately 60%, thanks to innovations in early diagnosis and treatment.^3^

It is observed that approximately 25 thousand men are diagnosed with prostate cancer every year in Turkey. However, due to inadequate prostate cancer screening, the majority of them are not diagnosed. One in every four men diagnosed with cancer every year around the world has prostate cancer, and in Turkey, only one in every 12 men on average is diagnosed with prostate cancer. Approximately 700 thousand men are diagnosed with prostate cancer every year in the USA and 350 thousand men in EU countries. Due to increased awareness on the subject, screening tests being performed more widely is the most important factor in increasing the number of people diagnosed.^3^

Prostate cancer is the second cause of cancer-related death among men in the United States. In a systematic review study, it was determined that prostate-specific antigen (PSA) screening can reduce the risk of prostate cancer death. In the same study, it is stated that it can lead to false positive results, biopsy complications, and overdiagnosis. Inferior effects of active treatments on long-term survival in prostate cancer detected by screening have been found.^4^

In 2021, the European Association of Urology (EAU) for the use of PSA testing has introduced new recommendations. These recommendations are risk-adjusted early prostate cancer detection recommendation that includes PSA testing, risk calculators, and parametric magnetic resonance imaging. Thus, it is aimed to distinguish important prostate cancer from insignificant prostate cancer.^5^-^7^ Family history, genetic predisposition, being over 50 years of age, eating habits that are high in saturated fat, carbohydrates, and red meat, and exposure to certain chemicals are considered risk factors for prostate cancer.^8^

In most studies, it is recommended that the individual decision of men aged 55-69 should be prioritized in the application of the periodic-based PSA screening test for prostate cancer and should be evaluated together with the clinician. Evaluation of the benefits and harms of screening, additional tests, false positives in the prostate biopsy, overdiagnosis, and treatment complications, treatment complications such as incontinence and erectile dysfunction should be considered. Studies also do not recommend PSA-based screening over 70 years of age.^9^,^10^

In another study, clinician consultation is recommended in which population screening in asymptomatic men is not recommended.^11^ While no difference was found in prostate cancer mortality over 10 years between non-screening standard practice and PSA screening, it was reported that screening reduced the number of low-risk prostate cancer cases.^12^ A population-based prostate cancer screening is not recommended in most countries. On the other hand, it is recommended to perform a PSA test and referral to a urologist as a result of the patient’s request and joint evaluation with the physician due to symptoms.^13^

Prostate diseases that threaten men’s health in the world and Turkey need to be identified early and sufficiently. It is important to determine awareness levels and reveal the current situation. In our study, we aimed to evaluate the results of the prostate-specific antigen (PSA) test used in prostate cancer screening in men aged 40 and over registered in a family health center.

## METHODS

The study was planned as field research with a retrospective pattern. In the study, the data of 1106 patients who applied to the family health center between January 2018 and May 2023 were evaluated. After the patients’ data from previous years were obtained from the records, individuals who met the study criteria were included. In this context, PSA levels in patients of different age categories and available data are examined in a retrospective study to determine risk factors for a particular disease. Referral to a urologist based on PSA results and prostate findings were also scanned from the records and included in the data.

The study was planned to determine the awareness level of prostate cancer screening in Turkish society and to reveal the current situation; We aimed to evaluate the results of the prostate specific antigen (PSA) test used in prostate cancer screening in male individuals aged 40 and over registered to a family health center.

The patients were examined according to their PSA levels according to their age, marital status, education, smoking and alcohol use, family history of prostate disease, genetic disease diagnosis in the patient, cancer diagnosis in the patient, number of comorbidities, and referral results. Patients were referred to a urologist, and their level of agreement with prostate cancer findings was examined according to the PSA results of the patients according to these groups. In addition, since it is known that patient ages have an impact on prostate cancer, PSA and diagnosis levels according to age were also examined in three different age categories.

The Complete census method was used as the sampling method in the research. Data regarding patients who met the study acceptance criteria were subjected to analysis. In this context, n=1106 patients, male, over 40 years old, who applied to the family health center between 2018-2023 and were screened with a PSA test, constitute the sample of the study. Since the data obtained in the study were obtained from records, according to the power analysis performed in the calculations of sample adequacy, it was seen that the power of the sample was 0.99 and the effect size level of the study was 0.53. In addition, according to the sampling methods, it has been observed that the current sample can explain the entire male population in Turkey with a 1% error and 95% level.

This retrospective study included 615 people (55.6%) aged 40-54, 379 people (34.3%) aged 55-69, and 112 individuals (10.1%) aged 70 and over. In the study, all data of male individuals aged 40 and over who were registered to the Family Health Center between 1-30 June 2023 were obtained from the database retrospectively. In the study, prostate-specific antigen (PSA) blood test results were performed on a total of 1106 male individuals aged 40 and over registered in the Uludag University Family Health Center, Bursa, Turkey during the five years between June 1, 2018, and May 31, 2023, were taken from the database and analyzed. Age, marital status, education level, occupation, smoking and alcohol use, family history of cancer, presence of prostate disease, concomitant diseases, prostate-specific antigen blood test (PSA, ng/mL) laboratory results, referral status to the urologist were determined, and recorded. The study was done anonymously. The data were evaluated by statistical analysis after the approval of the Ethics Committee.

### Ethical approval:

This retrospective study was performed after receiving the approval of the Clinical Research Ethics Committee of Bursa Uludag University (Reference No.: 2023-11/33, dated: May 16, 2023) and by the Declaration of Helsinki.

### Statistical analysis:

In the study, evaluation of the measurements was made as mean, standard deviation, frequency, and percentage. An independent sample t-test was used to examine the sub-dimensions of PSA measurements for variables with a two-stage group (Occupation, smoking, family history of cancer, cancer diagnosis). Sidak test was applied as an analysis of variance and dual follow-up test in the examinations performed in groups with three levels and above (Education, number of diseases, referral, age, etc.). P values less than 0.05 were considered statistically significant in the study. Analyzes were made with SPSS 25.0 package program.

## RESULTS

Approximately 15.5% of the participants in the study were married, 78.4% were single and 6% were separated from their spouses or widowed. According to the age of the participants, 55.6% were 40-54 years old, 34.3% were 55-69 years old, and 10.1% were 70 years and older. It was observed that the ages of the participants included in the study were 54.23±11.43 (40-93). The age distribution of the groups was 40-54 years old with 55.6%, 55-69 years old with 34.3%, and over 70 years old with 10.1%.

Education levels of 3.1% were below high school, 34.9% of them were at high school and 61.9% of them were at university level. It was determined that 82.9% of the participants did not smoke and 17.1% quit smoking. It was determined that 98.22% of the participants did not use alcohol and 1.8% were social drinkers. It was determined that the rate of industrial substance exposure was 0.2%, the rate of having a family history of prostate disease was 1.4%, the rate of being diagnosed with a genetic disease was 8.4%, and the rate of having another cancer diagnosis was 3.6%.

The rate of no comorbidity in the patient was found to be 38.6%. In addition, it was observed that 27.8% of the patients had one, 17.3% of them two, 12.2% of them three, and 3.7% of them had four different comorbid diseases. While 31.7% of the patients with prostate-related symptoms and referred to the urologist had no findings, 66.7% were diagnosed with benign prostatic hypertrophy and 1.6% with prostate cancer, (Table-I).

**Table-I:** Demographic features.

PARAMETERS		n	%
Marital status	Married	151	15.5%
	Single	766	78.4%
	Widowed or separated from spouse	59	6.0%
Education level	High school and below	30	3.1%
	High school	340	34.9%
	University	604	61.9%
Occupation	No	970	99.8%
	Industrial substance exposure	2	0.2%
Smoking	Never used	661	82.9%
	Left	136	17.1%
Alcohol	Never used	782	98.2%
	Social drinker	14	1.8%
Family history of prostate disease	No	771	98.6%
	Yes	11	1.4%
Genetic disease diagnosis	No	716	91.6%
	Yes	66	8.4%
Cancer diagnosis	No	754	96.4%
	Yes	28	3.6%
Number of comorbidities	No	301	38.6%
	1	217	27.8%
	2	135	17.3%
	3	95	12.2%
	4	29	3.7%
	5	1	0,1%
	6	2	0.3%
Result of referral to a urologist	No	80	31.7%
	Benign prostatic hypertrophy	168	66.7%
	Prostate cancer	4	1.6%
Age group	40-54 years	615	55.6%
	55-69 years	379	34.3%
	70 years and older	112	10.1%

The PSA values of the patients referred to the urologist were 1.21±0.98 ng/mL for the 40-54 years old, 5.75±4.71 ng/mL for the 55-69 years old, and 7.15±8.43 ng/mL for the over 70 years old (p=0.01). It was found that the PSA value determined for referral in the 40-45 age group was 1.21±0.98 ng/mL and lower than the other groups, (Table-II).

**Table-II:** PSA (Prostate-specific antigen) values by age.

Age group	Referral to urologist	p- Value

	Patient’s PSA value (ng/mL)	

	X±s.s.	
40-54 years	1.21±0.98	0.01*
55-69 years	5.75±4.71	
70 years and older	7.15±8.43	

**Analysis of variance

* Significant difference at the 0.05 level.

It was determined that the PSA levels of the patients referred to the urologist did not differ significantly in the 40-54 age group, in the patients with benign prostatic hypertrophy and without symptoms (p=0.23). No cancer was observed in this group.

It was further determined that the PSA levels of the patients referred to the urologist were significantly different in the 55-69 age group with benign prostatic hypertrophy and those without symptoms (p=0.01). The PSA levels of the patients with benign prostatic hypertrophy or prostate cancer were higher than the patients without any finding, and the mean was 6.81±4.39. 2 patients with cancer in this group were included in the benign group for comparisons.

In the group over 70 years of age, the mean PSA value was 7.05±8.48, and a finding was found in all patients. Two patients with cancer in this group were evaluated together with the other group, (Table-III). PSA levels were different according to marital status, and PSA levels were higher in singles and widows (p=0.01). According to the referral results, it was observed that the rate of finding symptoms was higher in the widowed and unmarried groups.

**Table-III:** Comparison of PSA values with results according to age.

Year	Referral result	Yes	p- Value

		Patient’s PSA value (ng/mL)	

		X±s.s.	
40-54 years	No	1.05±0.88	0.23
	Benign prostatic hypertrophy	1.50±0.98	
55-69 years	No	1.3±1.44	0.01*
	Benign prostatic hypertrophy - Prostate cancer	6.81±4.39	
70 years and older	Benign prostatic hypertrophy - Prostate cancer	7.05±8.48	-

**T-test analysis

* Significant difference at the 0.05 level.

We also found that PSA levels were different according to educational status, and PSA levels were higher in patients who graduated from high school (p=0.01). According to the referral results, the incidence of cancer was found to be higher in the groups that graduated from high school. Although the PSA level does not differ according to smoking status, the incidence of cancer and benign prostatic hypertrophy is lower in non-smokers (p=0.58). Furthermore, PSA level was different and higher in patients with a family history of prostate cancer. The incidence of cancer was higher in the group with a family history of prostate cancer (p=0.01). It was observed that PSA levels were not different according to the diagnosis of genetic disease in the patient (p=0.23).

It was observed that PSA levels were not different according to the presence of another cancer diagnosis in the patient (p=0.30). However, it has been observed that the rate of prostate cancer is higher in patients with a diagnosis.

We also noted that the PSA level was different according to the comorbidity status of the patient. In the study, it was observed that PSA levels were higher in patients with two or more diseases. The incidence of cancer was higher in the group with 3-4 diseases. It was determined that the rates of benign prostatic hypertrophy were higher in groups with 4-5-6 diseases (p=0.01).

According to the result of the referral to the urologist, PSA levels were different in patients with PSA values of six and above, while PSA levels did not differ in groups with benign prostatic hypertrophy or prostate cancer (p=0,01), (Table-IV).

**Table-IV:** Referral result and PSA compliance by demographic characteristics.

PARAMETERS				Referral result		

	Referral to urologist - Yes			No	Benign prostatic hypertrophy	Prostate cancer

	Patient’s PSA value (ng/mL)			%	%	%

		X±s.s.s	p- Value			
Marital status	Married	1±1	0.01*	100.0%	0.0%	0.0%
	Single	5±30		29.2%	69.4%	1.4%
	Widowed or separated from spouse	4±8		29.6%	66.7%	3.7%
Education level	High school and below	2±2	0.01*	45.5%	54.5%	0.0%
	High school	8±4.2		21.1%	74.7%	4.2%
	University	2±4		37.7%	62.3%	0.0%
Smoking	Never used	5.0±3.1	0.58	30.3%	68.7%	1.0%
	Left	4.02±7.2		37.3%	58.8%	3.9%
Alcohol	Never used	5.2±2.8	0.01*	30.9%	67.5%	1.6%
	Social drinker	1.52±0.23		66.7%	33.3%	0.0%
Family history of prostate disease	No	4.2±2.8	0.04	32.1%	66.7%	1.2%
	Yes	10.52±1.2		16.7%	66.7%	16.7%
Genetic disease diagnosis	No	5±3	0.23	32.6%	65.6%	1.8%
	Yes	3±5		25.0%	75.0%	0.0%
Cancer diagnosis	No	5.12±2.9	0.30	34.2%	65.4%	0.4%
	Yes	4.2±7.4		0.0%	83.3%	16.7%
Number of comorbidities	No	2.3±1.6	0.03*	68.8%	31.3%	0.0%
	1	2.5±2.2		29.2%	69.2%	1.5%
	2	8.1±5.3		36.2%	63.8%	0.0%
	3	7.2±6.1		20.0%	76.9%	3.1%
	4	7.3±9.2		5.6%	88.9%	5.6%
	5	8.4±0.5		0.0%	100.0%	0.0%
	6	13.2±3.5		0.0%	100.0%	0.0%
Result of referral to a urologist	No	1.25±1.3	0.01*	100.0%	0.0%	0.0%
	Benign prostatic hypertrophy	6.5±3.4		0.0%	100.0%	0.0%
	Prostate cancer	6.0±7.1		0.0%	0.0%	100.0%

**Analysis of variance

* Significant difference at the 0.05 level.

## DISCUSSION

It was observed in this study that the age of the group examined in the study was 54.23±11.43, which was in the prostate cancer risk group. However, for the patients examined, it was observed that the most at-risk group over the age of 70 was around 10%. This situation can be seen as an indicator that possible prostate cancer patients cannot be controlled. It was observed that the rate of referral to a urologist in the group over 70 years of age was 22.3%, and the rate of receiving a diagnosis as a result of referral was 33.1% in the entire group. It is thought that patients in this older age group, who are at risk in this care, are not diagnosed. In our study, we determined that 1.6% of men had been found with prostate cancer. Also interestingly, PSA levels did not differ in groups with benign prostatic hypertrophy or prostate cancer.

In a study evaluating the geographical distribution, epidemiological differences, and disease-related risk factors of prostate cancer in the world, it was determined that the prostate cancer incidence was highest in Northern Europe and the Caribbean, and lowest in South-Central Asian countries. It was determined that metabolic syndrome, the presence of concomitant disease, smoking, and obesity increased deaths from prostate cancer. It is stated that modifiable risk factors may affect the risk of dying from the disease, but there is not enough evidence that there is any clear indication for prevention other than early detection to reduce prostate cancer mortality.^11^ Another study reported that tobacco smoking, alcohol use, and a family history of cancer were associated with an increased risk of prostate cancer.^14^

In our study, the PSA level was different according to comorbidity status. PSA levels were higher in patients with two or more diseases, while cancer incidence was higher in patients with 3-4 diseases. In addition, those with more than four diseases had a higher incidence of benign prostatic hypertrophy. While there was no difference between PSA levels according to smoking, the incidence of cancer and benign prostatic hypertrophy was lower in never-smokers.

In addition to the determination of serum PSA levels by screening, the determination of free PSA levels is important in the urology clinic. Serum PSA and free PSA levels have been shown to decrease significantly, especially after combined treatment of advanced prostate cancers with hormone and radiotherapy.^15^ In another study, it is suggested that urinary PSA can complement serum PSA as an auxiliary marker in detecting particularly aggressive cancer.^16^ The diagnosis of benign prostatic hypertrophy and prostate cancer should be clearly defined in patients referred to the urology clinic. Unlike the diagnosis of cancer, the determination of hypertrophy is important in choosing the appropriate treatment.^17^ The primary contribution of the PSA screening results in our study to these procedures mentioned in urology clinics is important. Another study showed that PSA-based screening or general PSA testing is not a solution for this disease. It is anticipated that a risk-adjusted PSA screening could reduce unnecessary interventions.^18^ In our study, PSA levels and prostate cancer rates were found to be higher in patients with a family history of prostate cancer who were referred to a urologist.

In one study, prostate-specific antigen levels were associated with cancer-resistant patients’ vulnerability, economic and educational status, bone metastases, and symptoms.^19^ Therefore, in terms of primary health care, especially in elderly patients, the clinical status of the patient and prostate specific antigen levels should be evaluated first.

In a study conducted in England, the prostate-specific antigen (PSA) test was compared with the PSA test (opportunistic screening) performed in the absence of any symptoms. At the end of this study, despite its controversial results, it was determined that men preferred opportunistic screening. However, it is stated that this screening distorts the assessment of the rates in the general population.^20^

One study suggests two conditions for a screening program to be effective. The first is that the screening test determines the disease before the clinical diagnosis. The second is that there is a treatment that will prevent the related disease. It is stated that the discussions are effective in the diagnosis of PSA screening and related cancers.^21^

Although there are new genomic and technological opportunities to benefit from serum, urine, and tumor tissue in the diagnosis of prostate cancer, it is stated that these will also bring some difficulties. Therefore, the importance of an individualized approach in the early diagnosis of prostate cancer, classification of the disease, and response to treatment are emphasized.^22^

### Limitations:

**T**he group examined in this study was from Turkish society with data obtained from a family health center in the period between 2018 and 2023. The age of the group included in the study was 54.23±11.43. The marital status was predominantly single and the education level was predominantly at the undergraduate level. Alcohol and smoking, disease history, and comorbidity rates were found to be low. The patients included had a low risk of prostate cancer These must be kept in mind while interpreting the results of this study and findings cannot be generalized.

## CONCLUSION

It is important to diagnose benign prostatic hypertrophy or prostate cancer in patients aged 55-69 years, whose mean PSA levels were 6.81±4.39, and referred to a urologist. As a contribution to the discussions in the literature, we suggest that the inclusive approach of the family physician, who makes decisions together with the patient, reduces the uncertainties about the test. We emphasize the importance of the patient’s request and the family physician’s referral to the urologist due to symptoms in screening with the PSA test.

### Authors’ Contribution:

**OG:** Conceived, designed, did data collection, did statistical analysis & editing of the manuscript, manuscript writing, did review and final approval of the manuscript.

## References

[1] Sung H, Ferlay J, Siegel RL, Laversanne M, Soerjomataram I, Jemal A (2021). Global Cancer Statistics 2020:GLOBOCAN Estimates of Incidence and Mortality Worldwide for 36 Cancers in 185 Countries. CA Cancer J Clin.

[2] Sagan A, McDaid D, Rajan S, Farrington J, McKee M Screening:When is it appropriate and how can we get it right?Copenhagen (Denmark):European Observatory on Health Systems and Policies 2020. (Policy Brief, No. 35.).

[3] Aydin S (2007). Türkiye'de üriner sistem kanserlerin görülme sıklığı. Türk Üroloji Dergisi.

[4] Fenton JJ, Weyrich MS, Durbin S, Liu Y, Bang H, Melnikow J (2018). Prostate-Specific Antigen-Based Screening for Prostate Cancer:Evidence Report and Systematic Review for the US Preventive Services Task Force. JAMA.

[5] Van Poppel H, Roobol MJ, Chapple CR, Catto JWF, N'Dov J, Sønksen J (2021). Prostate-specific Antigen Testing as Part of a Risk-Adapted Early Detection Strategy for Prostate Cancer:European Association of Urology Position and Recommendations for 2021. Eur Urol.

[6] Poppel VH, Albreht T, Basu P, Hogenhout R, Collen S, Roobol M (2022). Serum PSA-based early detection of prostate cancer in Europe and globally:past, present and future. Nat Rev Urol.

[7] Magnani CJ, Li K, Seto T, McDonald KM, Blayney DW, Brooks JD (2019). PSA Testing Use and Prostate Cancer Diagnostic Stage After the 2012 U. S. Preventive Services Task Force Guideline Changes. J Natl Compr Canc Netw.

[8] Bergengren O, Pekala KR, Matsoukas K, Fainberg J, Mungovan SF, Bratt O (2023). 2022 Update on Prostate Cancer Epidemiology and Risk Factors-A Systematic Review. Eur Urol.

[9] Grossman DC, Curry SJ, Owens DK, Domingo KB, Caughey AB, Davidson KW, US Preventive Services Task For (2018). Screening for Prostate Cancer:US Preventive Services Task Force Recommendation Statement. JAMA.

[10] Ilic D, Djulbegovic M, Jung JH, Hwang EC, Zhou Q, Cleves A (2018). Prostate cancer screening with prostate-specific antigen (PSA) test:a systematic review and meta-analysis. BMJ.

[11] Jackson SD, Rue MRDL, Greenslade TP, John AM, Wahid S, Martin RM (2022). Screening asymptomatic men for prostate cancer:A comparison of international guidelines on prostate-specific antigen testing. J Med Screen.

[12] Martin RM, Donovan JL, Turner EL, Metcalfe C, Young GJ, Walsh EI (2018). Effect of a Low-Intensity PSA-Based Screening Intervention on Prostate Cancer Mortality:The CAP Randomized Clinical Trial. JAMA.

[13] Vickers A, O'Brien F, Montorsi F, Galvin D, Bratt O, Carlsson S (2023). Current policies on early detection of prostate cancer create overdiagnosis and inequity with minimal benefit. BMJ.

[14] Gandaglia G, Leni R, Bray F, Fleshner N, Freedland SJ, Kibel A (2021). Epidemiology and Prevention of Prostate Cancer. Eur Urol Oncol.

[15] Zhu H, Jiu T, Wang D (2015). Impact of polymorphisms of the DNA repair gene XRCC1 and their role in the risk of prostate cancer. Pak J Med Sci.

[16] Zhang S, Zhao S, Fu X (2019). Intensity modulated radiotherapy in combination with endocrinotherapy in the treatment of middle and advanced Prostatic Cancer. Pak J Med Sci.

[17] Höti N, Lih TS, Dong M, Zhang Z, Mangold L, Partin AW (2023). Urinary PSA and Serum PSA for Aggressive Prostate Cancer Detection. Cancers (Basel).

[18] Chang Y, Chang J, Wang H (2018). Transurethral balloon dilatation of the Prostate and Transurethral Plasmakinetic resection of the Prostate in the treatment of Prostatic Hyperplasia. Pak J Med Sci.

[19] Lakes J, Arsov C (2019). PSA-Screening und molekulare Marker (PSA screening and molecular markers). Urologe A.

[20] Feng J, Sun Q, Li J, Li TT (2021). Reliability and validity test of VES-13 and analysis of influencing factors for the vulnerable condition of patients with advanced castration-resistant prostate cancer. Pak J Med Sci.

[21] Clift AK, Coupland CA, Cox HJ (2021). Prostate-specific antigen testing and opportunistic prostate cancer screening:a cohort study in England, 1998-2017. Br J Gen Pract.

[22] Albertsen PC (2023). PSA testing, cancer treatment, and prostate cancer mortality reduction:What is the mechanism?. Urol Oncol.

